# Intraoperative near‐infrared II window fluorescence imaging‐assisted nephron‐sparing surgery for complete resection of cystic renal masses

**DOI:** 10.1002/ctm2.604

**Published:** 2021-10-14

**Authors:** Caiguang Cao, Shaohui Deng, Binshuai Wang, Xiaojing Shi, Liyuan Ge, Min Qiu, Fan Zhang, Min Lu, Lulin Ma, Chongwei Chi, Zhenhua Hu, Jie Tian, Shudong Zhang

**Affiliations:** ^1^ Beijing Key Laboratory of Molecular Imaging The State Key Laboratory of Management and Control for Complex Systems Institute of Automation Chinese Academy of Sciences Beijing China; ^2^ School of Artificial Intelligence University of Chinese Academy of Sciences Beijing China; ^3^ Department of Urology Peking University Third Hospital Beijing China; ^4^ Department of Pathology Peking University Third Hospital Beijing China; ^5^ Beijing Advanced Innovation Center for Big Data‐Based Precision Medicine School of Medicine Beihang University Beijing China; ^6^ Engineering Research Center of Molecular and Neuro Imaging of Ministry of Education School of Life Science and Technology Xidian University Xi'an China

Dear Editor,

The resection of cystic renal masses (CRMs) represents a challenging process in terms of nephron‐sparing surgery because the mostly fluid‐filled growth pattern of renal cancers causes them easy to rupture.^1^ As the malignant tumours rupture, cancer cells metastasize in the abdominal cavity, patients face a high risk of recurrence.^2^ Meanwhile, studies have shown that more functional nephrons correlate with a better prognosis of patients.^3^ Therefore, it is crucial to achieving accurate and complete tumour resection during partial nephrectomy (PN).

The near‐infrared II (NIR‐II, 1000–1700 nm) window fluorescence imaging is a highly promising strategy for medical applications. Based on our experience of image‐guided surgery,^4^ a novel NIR‐II fluorescence imaging system was developed for helping the resection of CRM (Supplementary S1). From October 2019 to November 2020, nine patients who underwent PN for resection of CRM were enrolled (Table 1). After exposing tumours, indocyanine green (ICG, 0.5 mg/kg body weight) was injected intravenously, the NIR‐II fluorescence imaging system was used for the visualization of tumours (Figure 1, Supplementary S2). Under the guidance of NIR‐II fluorescence imaging, all tumours were completely resected without rupture, no recurrence or metastasis was found after 4–17 months of follow‐up.

**TABLE 1:** Characteristics and preoperative diagnosis of patients with CRM

Variable	Value
Patients, *n*	9
Age/year, M (range)	46 (35–65)
Gender, *n*	
Male	8
Female	1
Affected side, *n*	
Left	5
Right	4
Tumour diameter/mm, M (range)	48 (27–61)
Preoperative eGFR, M (range)	92 (68–113)
RENAL score, M (range)	8 (4–9)
Ischaemia time/min, M (range)	14 (10–25)
Blood loss/ml, M (range)	100 (10–400)
Hospital stay/day, M (range)	6 (3–6)
Pathology	
ccRCC	4
BRC	4
PKD	1
Postoperative eGFR, M (range)	72 (41–105)

Abbreviations: BRC, benign renal cyst; ccRCC, clear cell renal cell carcinoma; eGFR; estimated glomerular filtration rate; M, median; PKD, polycystic kidney disease.

**FIGURE 1:**
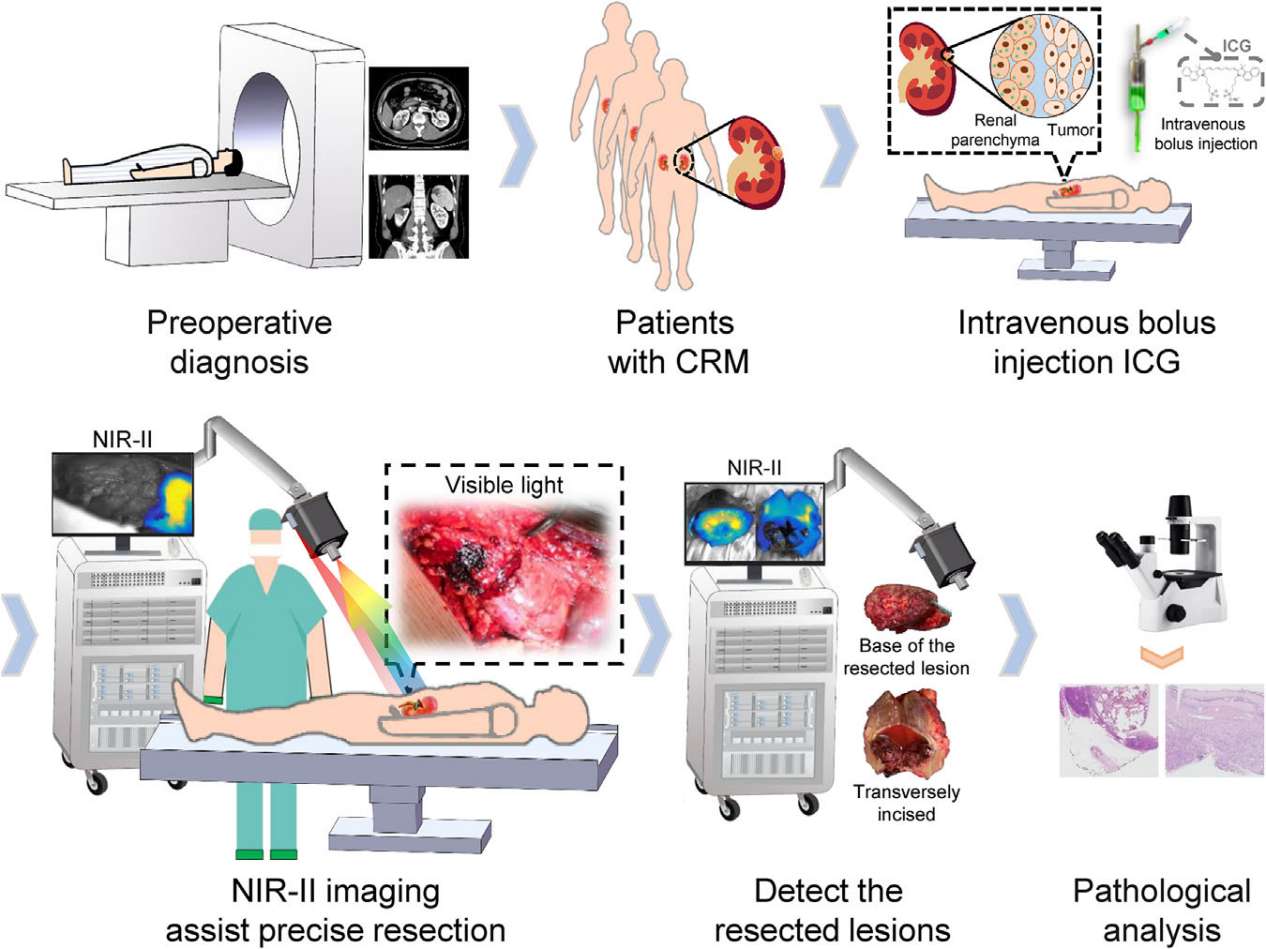
Flow diagram of the study protocol. Patients first received preoperative imaging examinations to determine whether eligible for the study. On the day of surgery, after laparotomy, ICG with a dose of 0.5 mg/kg body weight was administrated intravenously before blocking the renal artery. Then, the tumour boundaries were marked on the kidney surface under the guidance of the NIR‐II images, which were followed by arteries clamping and tumour resection. After resection, images of the surgical margins and resected lesions in the white‐light illumination and NIR‐II region were acquired separately. Lastly, pathological examinations were carried out

For a representative patient, the enhanced CT examination revealed a renal mass located at the right kidney (Figure 2A and B). After kidney exposure, the surgical region was first imaged under white light illumination (Figure 2C). One minute after ICG injection, NIR‐II fluorescence was observed in renal parenchyma and partial of the tumour vessels. The boundaries between tumour and renal parenchyma were distinct in the NIR‐II fluorescence images (Figure 2D and E), in which the renal parenchyma‐to‐tumour (RP/T, defined in Supplementary S3) fluorescence intensity ratio was 6.37 and the contrast‐to‐noise ratio (CNR, defined in Supplementary S3) was 5.25. Subsequently, tumour boundaries were marked by electrocautery guided by NIR‐II imaging (Figure 2F). Fluorescence signal intensity extracted from the position of the black arrow in Figure 2E showed the intensity weakened sharply from renal parenchyma to tumour (Figure 2G). The three‐dimensional fluorescence distribution map of the imaged area in Figure 2D further showed the signal intensity of renal parenchyma was significantly higher than that of the tumour (Figure 2H), which again proved the efficacy of NIR‐II imaging to detected tumour boundaries. After resection, the visual inspection did not detect residual tumours on the surgical margin (Figure 2I), the NIR‐II imaging also exhibited intense fluorescence (Figure 2J). A similar fluorescence‐guided surgery was conducted on other patients (Figure S1 and Video S1).

**FIGURE 2:**
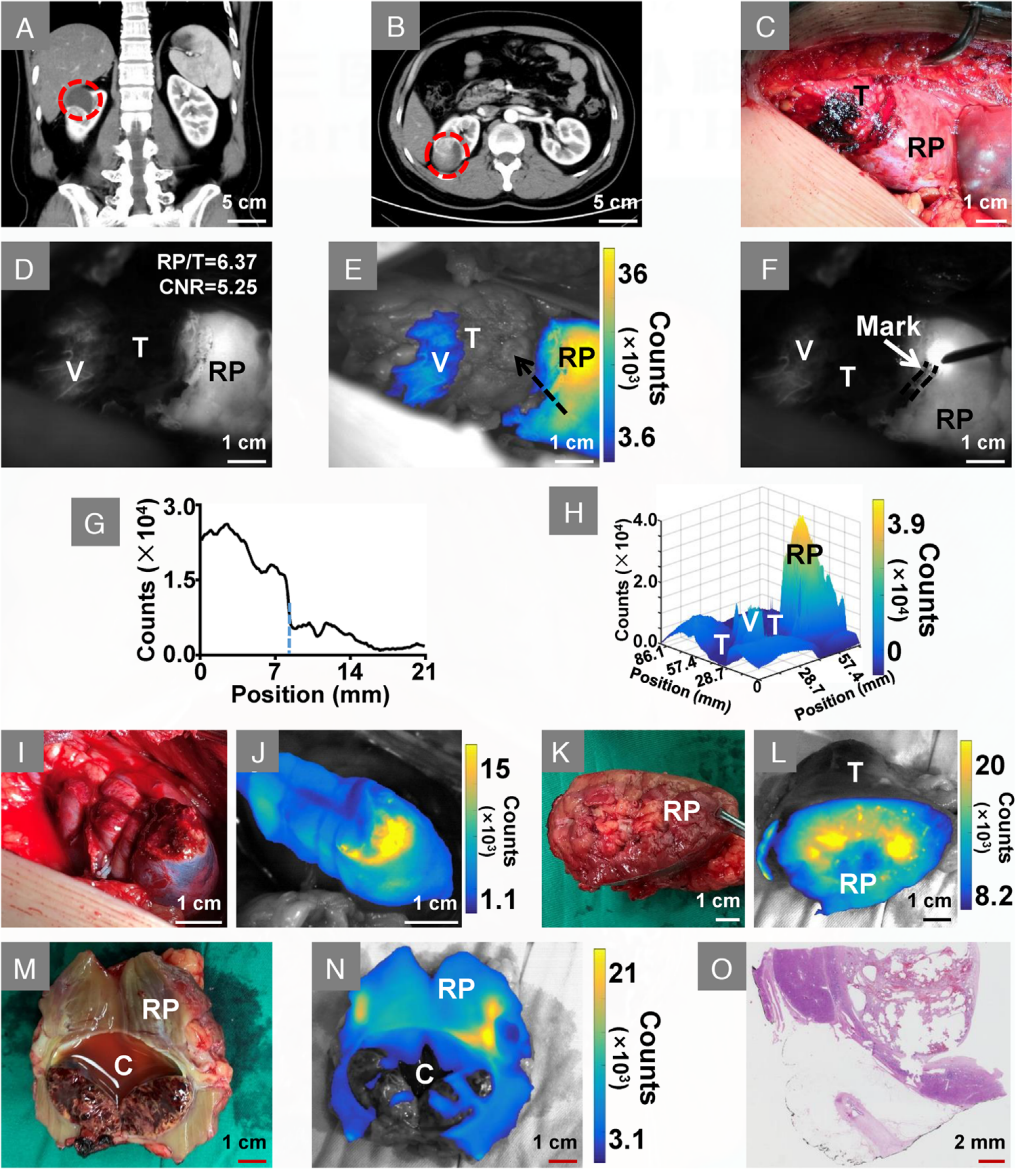
Intraoperative NIR‐II image‐assisted tumour resection. (A and B) Coronal‐plane and transverse‐plane of the enhanced CT image, red circle indicates a renal mass located at the right kidney. (C) Visible‐light image of the tumour and renal parenchyma. (D) Fluorescence is observed in renal parenchyma and partial tumour vessels after ICG injection. (E) Overlay image of D, in which fluorescence is shown in pseudo‐colour. (F) Tumour boundaries were marked by electrocautery on the kidney surface, the black dotted line showed the mark traces. (G) Cross‐sectional fluorescence intensity, which was extracted from the position of the black arrow in E. The blue dotted line in G indicates the place where is the boundary between tumour and renal parenchyma. (H) Three‐dimensional mapping of the fluorescence distribution of D. (I) White‐light illumination image of the surgical margins. (J) NIR‐II imaging shows intense fluorescence on the surgical margin. (K) Visible‐light image of the tumour base. (L) Overlay image shows intense fluorescence on the base of the tumour. (M) Visible‐light image of the tumour cavity, the intact internal fluid indicates the tumour is resected without rupture. (N) Overlay image of the tumour cavity. (O) Pathological examination with haematoxylin and eosin staining shows the tumour is ccRCC. Abbreviations: C, tumour cavity; RP, renal parenchyma; T, tumour; V, vessel

Subsequently, resected tissue of the patient was further inspected, intense fluorescence was detected on the base of the lesion (Figure 2K and L). Combining the NIR‐II images of the surgical margin with the visual examination, the tumour was considered to be completely removed. The lesion was then dissected along its maximum axis, the internal fluid in the cavity indicated no rupture occurred during resection (Figure 2M and N). Finally, pathological examination showed the tumour was clear cell renal cell carcinoma (ccRCC) and was completely resected (Figure 2O).

The fluorescence images of the dissected lesions were further analysed. Quantitative analysis showed that NIR‐II imaging was able to identify ccRCC from the resected lesions (Figure 3, Figure S2). For a benign renal cyst transversely incised, almost no fluorescence was exhibited in the tumour cavity (Figure 3A–C). The cross‐sectional fluorescence intensity profile also showed a lower intensity in the tumour cavity (Figure 3D). Conversely, fluorescence was detected in some areas of the tumour cavity of ccRCC (Figure 3E–G), which was proved by the cross‐sectional fluorescence intensity profile (Figure 3H). Eight of the nine resected lesions were dissected and all the tumour cavity‐to‐background (C/B, defined in Supplementary S3) fluorescence intensity ratio of ccRCCs was much higher than those of benign tumours (Figure 3I and J; 4.87 ± 3.40 vs. 1.53 ± 0.15, *p *= 0.1405). Besides, for each ccRCC, the C/B ratio was greater than 2.0, while the C/B ratio was lower than 1.8 for benign tumours, the receiver operating characteristic analysis about categorizing resected lesions showed the area under the curve (AUC) was 1.00 (Figure 3K).

**FIGURE 3:**
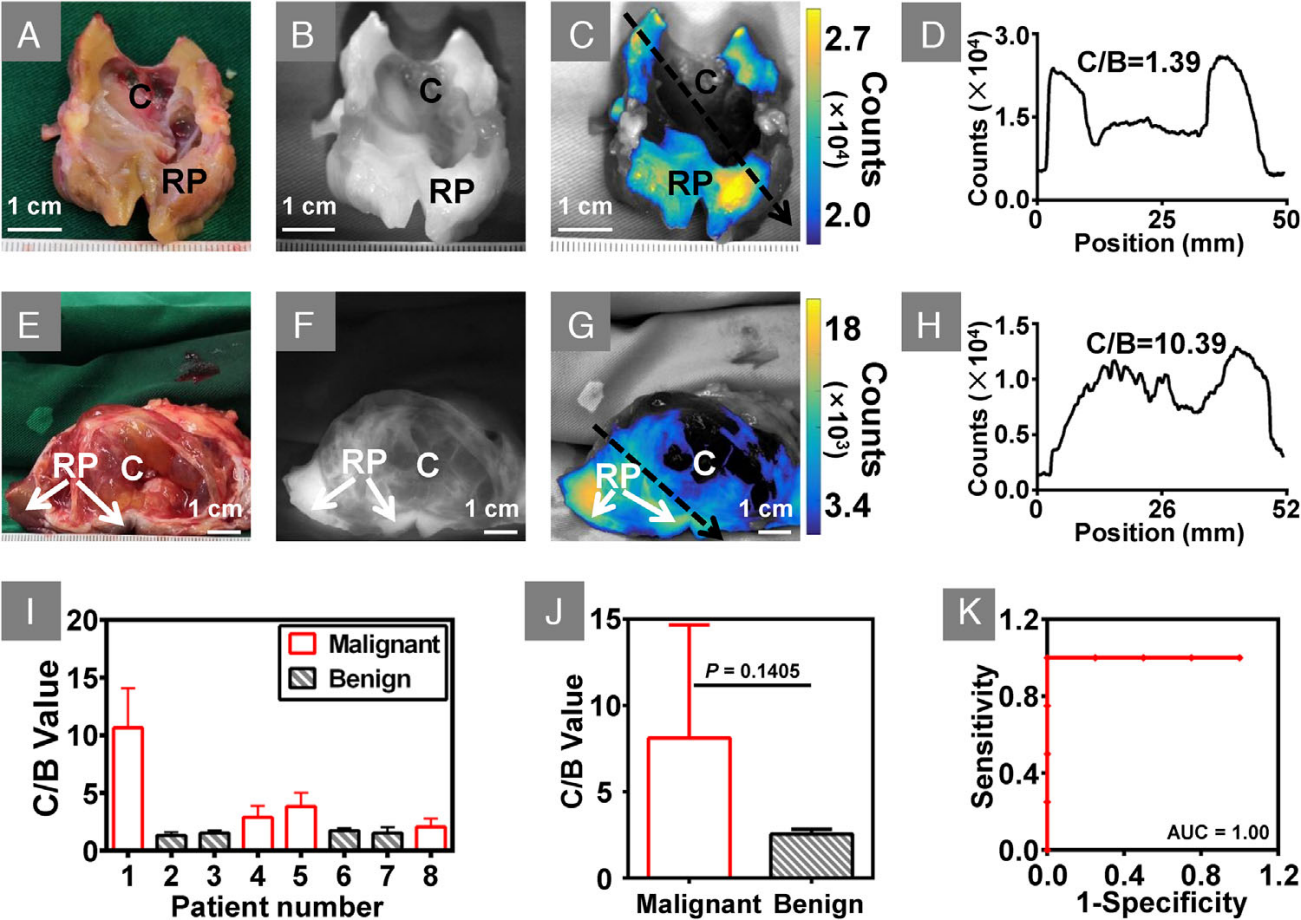
NIR‐II imaging identified ccRCC from resected lesions. (A) A benign renal cystic tumour was dissected along the maximum axis. (B and C) NIR‐II image and overlay image showed there was almost no fluorescence in the tumour cavity. (D) Cross‐sectional fluorescence intensity corresponding to the location and direction of the black arrow in C, which also demonstrated the lower intensity in the cavity. (E) A ccRCC tumour was dissected along the maximum axis. (F and G) NIR‐II image and overlay image showed some areas of the tumour cavity also appeared fluorescence. (H) Cross‐sectional fluorescence intensity corresponding to the location and direction of the black arrow in G, which also showed the intense fluorescence presented in the tumour cavity. (I) Bar graph of the C/B ratio of each patient, the error bars indicate the standard deviation over all pixels within the region of interest of tumour cavity. (J) The comparison of the average ratio of C/B between benign and malignant tumours. (K) The ROC analysis about discriminating ccRCC by NIR‐II imaging. Abbreviations: C, tumour cavity; RP, renal parenchyma

As shown in the above results, clinical benefits were brought with the use of NIR‐II imaging. ICG‐based traditional first near‐infrared window (700–900 nm) fluorescence imaging of renal tumour is limited to reduce the positive margin rate.^5^ The rate of positive surgical margins after nephron‐sparing surgery is as high as nearly 10%.^6^ One of the key obstacles is that those small residual tumours on the surgical margins are shadowed by the intense fluorescence of renal parenchyma during fluorescence imaging. In this study, surgical margins and base of the resected lesions received NIR‐II imaging, only when both appeared intense fluorescence was the tumour considered completely removed. Our pilot clinical results demonstrate this approach is an effective method for detecting residual tumours in the condition where normal tissue exhibits higher intense fluorescence than tumours.

The ICG injection dose is an important influence factor of fluorescence imaging during PN. Briefly, different dose causes renal parenchyma appears inadequate fluorescence or otherwise tumours appear undesirable fluorescence.^7^ Diverse injection doses have been reported, but not all tumours were successfully detected in the previous studies.^8^, ^9^ In this trial, ICG at a dose of 0.5 mg/kg body weight was administrated, benefited from the lower auto‐fluorescence and less interfered from the ambient light in the NIR‐II region, tumours were distinctly identified in all patients. The injection dose can be a reference for NIR‐II imaging in other renal‐related researches. Additionally, the ccRCC was identified based on the diversity of C/B ratios between different lesions. Although the relationship between the fluorescence in the tumour cavity and the ICG administration dose needed to be investigated. Our finding highlights the NIR‐II imaging as a potential diagnostic technique that may alter the treatment of CRM.

In summary, the first clinical trial of NIR‐II imaging for the resection of CRM was reported in this study. The novel technique brings benefits to patients, such as no positive surgical margins and no tumour ruptured during resection. In the future, other relevant benefits of the NIR‐II imaging assists CRM resection can be explored.

## CONFLICT OF INTERESTS

The authors declare no conflict of interests.

## Supporting information

Supplement information

Supplement information
