# Supplementary material for: Intraoperative near‐infrared II window fluorescence imaging‐assisted nephron‐sparing surgery for complete resection of cystic renal masses
Source: Clin Transl Med. 2021 Oct 14;11(10):e604. doi: 10.1002/ctm2.604 (PMC8516362; doi:10.1002/ctm2.604)
Supplement: Supplementary file 1 — Supplement information [file CTM2-11-e604-s002.docx]

**——Supplementary Materials——**

**Intraoperative near-infrared II window fluorescence imaging-assisted nephron-sparing surgery for complete resection of cystic renal masses**

Caiguang Cao^1,2,#^, Shaohui Deng^3,#^, Binshuai Wang^3,#^, Xiaojing Shi^1,2^, Liyuan Ge^3^, Min Qiu^3^, Fan Zhang^3^, Min Lu^4^, Lulin Ma^3^, Chongwei Chi^1^, Zhenhua Hu^1,2,*^, Jie Tian^1,2,5,^^6,*^, Shudong Zhang^3,*^

1. Beijing Key Laboratory of Molecular Imaging, The State Key Laboratory of Management and Control for Complex Systems, Institute of Automation, Chinese Academy of Sciences, Beijing, China

2. School of Artificial Intelligence, University of Chinese Academy of Sciences, Beijing, China

3. Department of Urology, Peking University Third Hospital, Beijing, China

4. Department of Pathology, Peking University Third Hospital, Beijing, China

5. Beijing Advanced Innovation Center for Big Data-Based Precision Medicine, School of Medicine, Beihang University, Beijing, China

6. Engineering Research Center of Molecular and Neuro Imaging of Ministry of Education, School of Life Science and Technology, Xidian University, Xi’an, China

# These authors contributed equally to this work.

* Corresponding Authors: Shudong Zhang, Department of Urology, Peking University Third Hospital, Beijing 100191, China.Phone: (+86)010-82267522; Fax: (+86)010-82020756; E-mail: shootong@163.com; Jie Tian, Institute of Automation, Chinese Academy of Sciences, Beijing 100190, China. Phone: (+86)010-82618465; Fax: (+86)010-62527995; E-mail: tian@ieee.org; Zhenhua Hu, Institute of Automation, Chinese Academy of Sciences, Beijing 100190, China. Phone: (+86)010-82628760; Fax: (+86)010-62527995; E-mail: zhenhua.hu@ia.ac.cn

**S1 Intraoperative NIR-II fluorescence imaging system**

To perform precise resection of CRM intraoperatively, a NIR-II fluorescence imaging system was constructed, which mainly consisted of a laser excitation unit and an imaging unit. The laser excitation unit mainly included a laser generator with a maximum power of 20w, a laser fiber, and a spherical glass beam expander. The laser power at the imaging site was adjustable. The wavelength of the excitation light was 792 nm. The beam expander was equipped at the end of the laser fiber to ensure that an excitation light with relatively homogeneous energy could illuminate the surgical region. The imaging unit was mainly composed of an InGaAs NIR-II camera (NIRvana 640, Teledyne Princeton Instruments) for acquiring NIR-II images and grayscale images, a high-performance lens (SWIRON 2.8/50, Schneider Kreuznach), and a long-pass optical filter (FELH1000, Thorlabs). The filter was coupled with the lens using a homemade adapter. Since the wavelength range responded by the NIR-II camera sensor was 900-1700 nm, the fluorescence signal with a wavelength of 1000-1700 nm was collected by this system. The fluorophore used in this study was ICG that with an emission peak of 820 nm. Thus the fluorescence signal at the emission wavelength tail of the agent was acquired. The emission spectrum of ICG at the excitation wavelength of 792 nm was shown in Fig. S3. Besides, the imaging unit also included a Huawei Honor V10 mobile phone for acquiring images under white light illumination. To obtain optimal imaging views, the NIR-II camera was fixed on a multi-axis robotic arm which was fixed on a wheeled base. The robotic arm, excitation equipment, and cameras were covered with a transparent sterile drape to reduce the risk of infection, the entire system could be easily moved and placed in the surgical room. The specific parameters about the optics of the system were presented in Table S1.

Besides, to process the acquired NIR-II images quickly, a specific Graphical User Interface based on MATLAB (MATLAB R2014b, The MathWorks, Inc.) was developed. NIR-II images and grayscale images were converted to overlay images based on the interface to simultaneously provide tissue information and a clear distribution of fluorescence. In detail, a threshold was first set through the interface, and pixels of NIR-II images with intensity below the threshold were not displayed. Other pixels whose intensity was higher than the value were set with blue color to yellow color based on the fluorescence intensity from low to high. The threshold was the average value of the fluorescence signal extracted from the tumor region in the NIR-II images, the tumor region was determined by the preoperative images. Then the produced pseudo-color image was overlaid with the grayscale image to produce an overlay image and was then displayed through the interface.

**S2 Patients’ characteristics and clinical protocol**

**S2.1 patients’ characteristics**

Patients were recruited from Peking University Third Hospital between October 2019 and November 2020. Nine patients with CRM were enrolled (Table S2). Inclusion criteria included age 18-80 years, tumors could be resected after clinical evaluation. Exclusion criteria were patient history of allergy to ICG or iodine, liver disease, pregnancy, or lactation. This study was conducted following the Declaration of Helsinki and was approved by the Peking University Third Hospital Medical Science Research Ethics Committee (IRB00006761-M2019404) and was registered at Chictr.org.cn (ChiCTR2000033471).

**S2.2 clinical protocol**

Patients first received enhanced CT scans and those with CRM and suitable for open surgery were enrolled. Once the patient signed informed ethics consent, the open PN was performed by the same surgeon through a flank approach. After incision of the Gerota’s fascia, the renal artery was exposed and the perinephric fat was removed with blunt or sharp dissection. The kidney surface was first imaged under white light illumination. Then ICG (Dandong Yichuang Pharmaceutical Co., Ltd.) was diluted to a concentration of 2.5 mg/ml using sterile water and was administered by intravenous bolus injection with a dose of 0.5 mg/kg within 10 seconds. One minute post-injection of ICG, NIR-II imaging was performed to determine the tumor boundaries, then the resection region was marked on the kidney surface under the guidance of NIR-II images followed by clamping arteries and tumor resection. During resection, the final resection margin was selected on the renal parenchyma and typically 3-5 mm away from the electrocautery mark.

After resection, NIR-II imaging on surgical margins and the base of the resected lesions was performed to detect the residual tumors. Then tumors were dissected along the maximum axis to determine whether the internal fluid was intact. Finally, all the resected lesions underwent pathological examination using hematoxylin and eosin staining, some also underwent immunohistochemical staining for further confirmation. The negative surgical margins were finally confirmed by postoperative pathological examination.

**S2.3 *In vivo* and *ex vivo* NIR-II imaging**

The imaging system was first initialized and covered with a transparent sterile drape before using and then placed next to the operation bed. For both *in vivo* and *ex vivo* imaging, the power density of the excitation light at the imaging site was set as 50 mW/cm^2^. To avoid the influence of the reflection from excitation light, an angle was reserved between the excitation path and the imaging path. The exposure time was set to 50 ms and it could be fine-tuned to obtain high-quality images. The working distance of the camera was adjusted to 50 cm for *in vivo* imaging. For *ex vivo* imaging of resected lesions, the multi-axis robotic arm was adjusted to guarantee the camera to get the best imaging field of view.

**S3 Analysis of NIR-II images and statistical analysis**

The fluorescence intensity of NIR-II images was extracted by ImageJ (1.8.0_112, National Institutes of Health, USA). The renal parenchyma-to-tumor (RP/T) fluorescence intensity ratio was defined as the ratio of mean signal intensity of renal parenchyma to that of the tumor. The contrast-to-noise ratio (CNR) was defined as (mean signal renal parenchyma – mean signal background) / standard deviation background^[1-2]^, the background was the tumor area. The cavity-to-background (C/B) fluorescence intensity ratio was defined as the ratio of mean signal intensity of tumor cavity to that of sterile cloth. Clinical results were presented as median and range, and the results of RP/T and C/B were expressed as mean ± s.d.). The difference of RP/T or C/B value between two groups of samples was assessed by the two-sided Welch’s *t*-test.

**S4 Discussion**

In this study, the NIR-II imaging was successfully applied for the guidance of CRM resection. No tumors ruptured during resection and no positive surgical margins were found by pathological examination. The main goal of the application of NIR-II imaging for guiding tumor resection has been achieved.

Quantitative results indicated that NIR-II imaging was able to identify ccRCC from the resected lesions. The mean C/B value of ccRCCs was 4.87 ± 3.40, and that for benign tumors was 1.53 ± 0.15. More importantly, the main role of NIR-II fluorescence imaging in this study was to identify the tumor boundaries and detect the residual tumors. After enhanced CT scans, the patients with cystic renal masses and meeting the inclusion criteria were included in this study, all tumors of the included patients should be resected after clinical evaluation. Regardless of whether the tumor was benign or malignant, the aim of all the surgery is consistent, that is completely resected the tumor while persevering more renal parenchyma. Thus, the judgment of benign and malignant for resection tissues would not change the surgical strategy. The discrimination results of NIR-II imaging were an additional function alongside fluorescent navigation. Although the *P*-value was 0.1405 which was not statistically significant and only marginal differences were presented at present, it also provided an auxiliary method for surgeons to distinguish benign and malignant tumors during surgery. The NIR-II imaging may bring potential value for the treatment of CRM in the future.

A major limitation of ICG is its short half-life in the blood^[3-4]^. In this study, one minute after injection of ICG, fluorescence was observed in renal parenchyma. Then the tumor boundaries were marked by electrocautery under the guidance of NIR-II imaging. The mean duration of marking boundaries was about 3 min for the nine presented patients. Additionally, tumors were resected with the arteries of the kidney blocked, ICG accumulated in the kidney throughout the resection. The renal parenchyma still displayed intense fluorescence after tumor resection. Thus, despite the short half-life, it did not impact fluorescence detection during the whole surgery.

Besides, the tail of the ICG emission spectrum in the NIR-II region was detected in this study. Our previous study has demonstrated that NIR-II imaging showed shining performance than NIR-I imaging under the same conditions^[5]^. Similarly, the high effectiveness of NIR-II imaging was also achieved in this study. Intense fluorescence in the NIR-II region was detected using our ICG dose, exposure time, laser power, etc. Although the emission intensity of ICG in the NIR-II region is weaker than that in the NIR-I region, the significant difference in signal intensity between the renal parenchyma and tumor was sufficient for tumors detection and boundaries identification.

All in all, the NIR-II imaging has been successfully applied for the resection of CRM. The high performance demonstrates it can be employed in other renal tumor resections in the future.

**Reference**

1. Tichauer KM, Samkoe KS, Sexton KJ, Gunn JR, Hasan T, Pogue BW. Improved tumor contrast achieved by single time point dual-reporter fluorescence imaging. *J Biomed Opt.* 2012;17(6):066001.
2. Hoogstins C, Burggraaf JJ, Koller M, et al. Setting Standards for Reporting and Quantification in Fluorescence-Guided Surgery. *Mol Imaging Biol.* 2019;21(1):11-18.
3. Li B, Zhao M, Feng L, et al. Organic NIR-II molecule with long blood half-life for in vivo dynamic vascular imaging. *Nat Commun.* 2020;11(1):3102.
4. Park T, Lee S, Amatya R, et al. ICG-Loaded PEGylated BSA-Silver Nanoparticles for Effective Photothermal Cancer Therapy. *Int J Nanomedicine.* 2020;15:5459-5471.
5. Hu Z, Fang C, Li B, et al. First-in-human liver-tumour surgery guided by multispectral fluorescence imaging in the visible and near-infrared-I/II windows. *Nat Biomed Eng.* 2020;4(3):259-271.


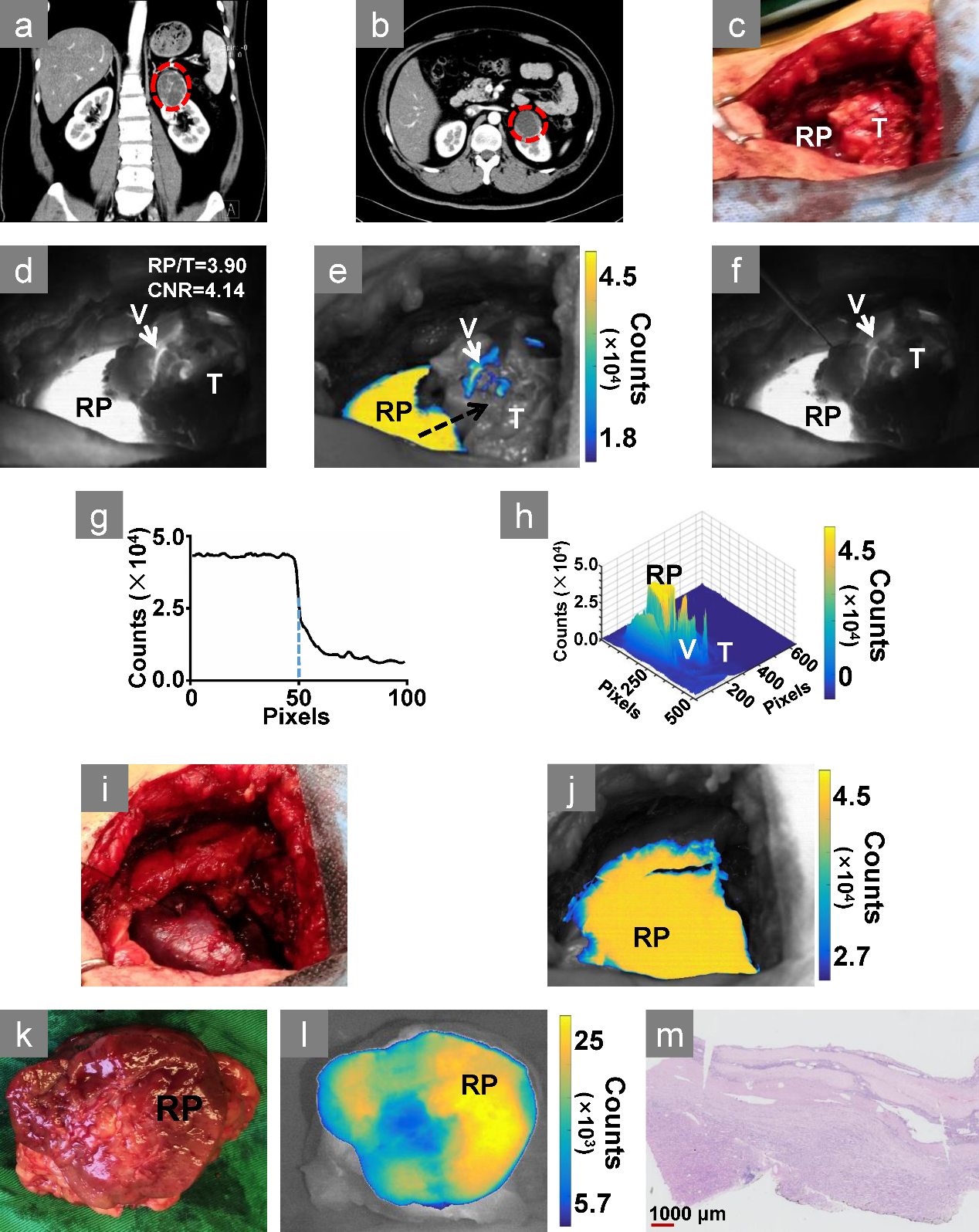


**Fig. S1.** Another patient underwent NIR-II image-assisted tumor resection. a-b, Red circle in the enhanced CT image of the coronal-plane and transverse-plane indicated a renal mass located at the left kidney. c, Visible light image of the tumor and renal parenchyma after laparotomy. d, NIR-II image was acquired after the injection of ICG, partial of the tumor vessels also appeared fluorescence. The RP/T and CNR were 3.90 and 4.14, respectively. e, Overlay image of d, in which fluorescence was shown in pseudocolor. f, Tumor boundaries were marked by electrocautery. g, Cross-sectional fluorescence intensity, which corresponded to the location and direction of the black arrow in e. The blue dotted line indicated that the signal intensity from renal parenchyma to the tumor weakened sharply. h, Three-dimensional mapping of the fluorescence distribution of d. i, Visible light image of the surgical margin. j, NIR-II imaging showed intense fluorescence on the surgical margin. k, The base of the resected tumor. l, NIR-II image showed intense fluorescence on the base of the tumor. m, Pathological examination with hematoxylin and eosin staining showed the tumor is ccRCC. T=tumor; PR=renal parenchyma; V=vessel;


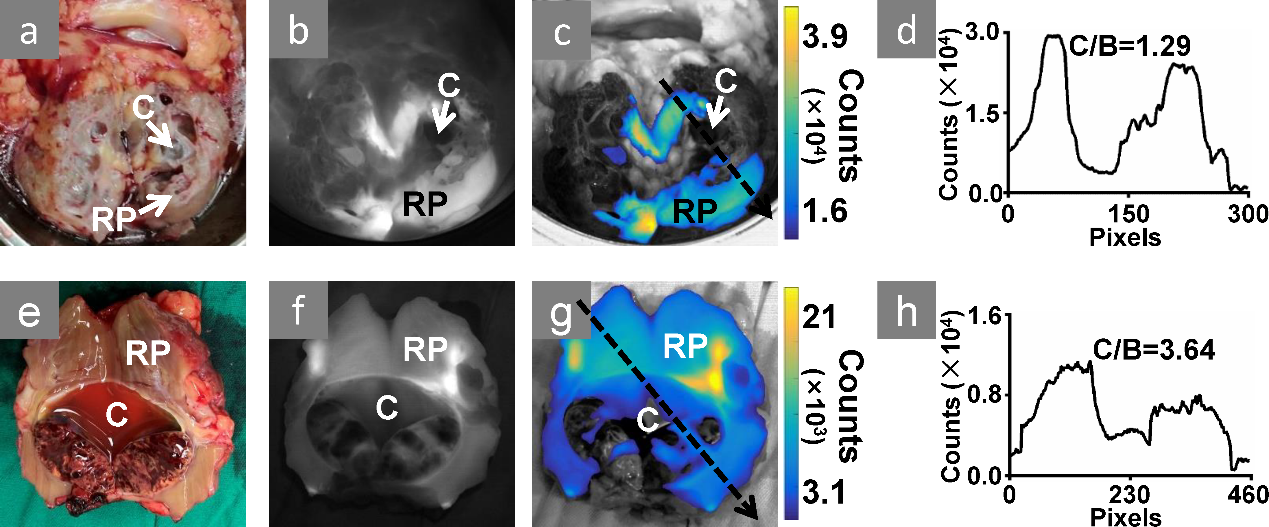


**Fig. S2.** Another example of NIR-II imaging identified ccRCC from resected lesions. a, Tumor cavity of a PKD tumor. b-c, NIR-II image, and overlay image, in which there was almost no fluorescence in the tumor cavity. d, Black arrow in c corresponded to the location and direction of cross-sectional fluorescence intensity that demonstrated the lower intensity in the tumor cavity. e, Tumor cavity of a ccRCC tumor. f-g, NIR-II image, and overlay image of tumor cavity. h, Black arrow in g corresponded to the location and direction of cross-sectional fluorescence intensity showed the intense fluorescence in the tumor cavity. RP=renal parenchyma; C=tumor cavity


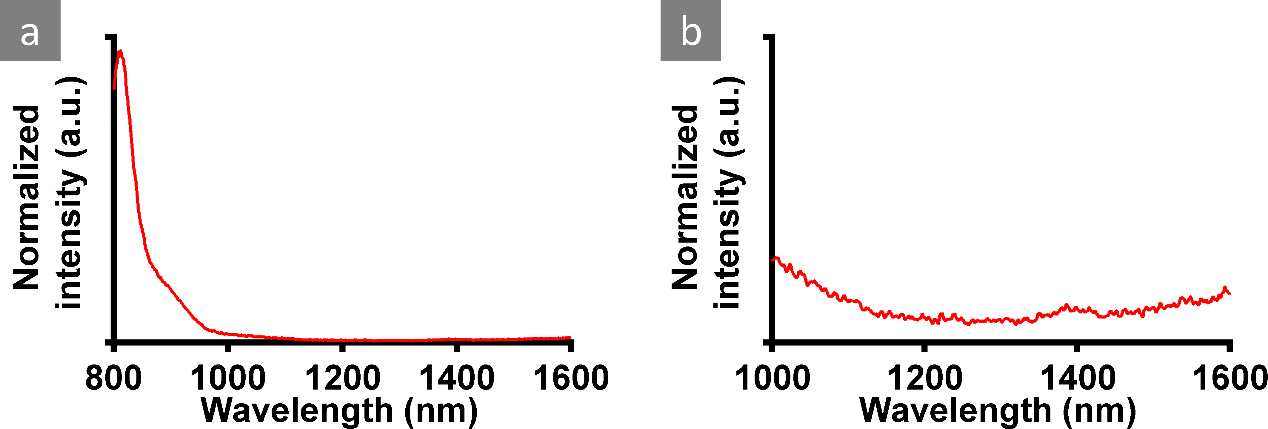


**Fig. S3.** The emission spectrum of ICG at the excitation wavelength of 792 nm. ICG was first dissolved in deionized water at the concentration of 1×10^-3^ mg/ml. Then the emission spectrum at the excitation wavelength of 792 nm has been recorded by the FLS980 Spectrometer (Edinburgh Instruments, Ltd., UK). a, the emission spectrum at 800-1600 nm. b, Ten times magnified view of the emission spectrum at 1000-1600 nm, the normalized intensity on the vertical axis in b is 0.1 times of a.

**Table S1.** Key parameters about optics of the developed intraoperative NIR-II fluorescence imaging system.

| **Instrument features** | **Parameters** |
| --- | --- |
| Imaging channel | NIR-II |
| Device type | NIRvana 640, Teledyne Princeton Instruments |
| Chip area (mm) | 12.80×10.24 |
| Highest temporal resolution  (ms) | 45.45 |
| *In vivo* working distance (cm) | 50 |
| Surgical field of view (cm) | 10.7×8.5 |
| Sensor resolution  (imaging pixels) | 640×512 |
| Imaging resolution (µm) | 62.5 |
| Sensitivity (ICG, fM) | 64.5 |
| Optical filter | 1000 nm long-pass, FELH1000, Thorlabs |
| Laser fiber | Customized, Made of quartz, 3 meter length |
| Beam expander | Customized, made of a spherical glass |
| Excitation power  at the imaging site | 50 mW/cm^2^ |
| Image windows | NIR-II; Visible (Color, Grayscale); Overlay |

**Table S2.** Patients’ characteristics and perioperative data.

| Patient  (n) | Age  (years) | Gender  (male  /female) | Bosniak classific-ation | Affected side  (left/right) | Tumor diameter  (mm) | Pre-eGFR | RENAL score | Ischemia  time (min) | Blood loss  (ml) | Hospital  stay  (d) | Pathology | ISUP  classification | Post-eGFR | Follow-up (month) |
| --- | --- | --- | --- | --- | --- | --- | --- | --- | --- | --- | --- | --- | --- | --- |
| 1 | 35 | M | III | Left | 52 | 113 | 7a | 11 | 50 | 5 | ccRCC | I | 92 | 17 |
| 2 | 61 | M | III | Left | 58 | 68 | 9a | 25 | 300 | 6 | PKD | - | 77.6 | 15 |
| 3 | 56 | M | IIF | Left | 43 | 92 | 8a | 10 | 100 | 6 | BRC | - | 53 | 15 |
| 4 | 46 | M | IV | Right | 48 | 105 | 8p | 18 | 100 | 6 | ccRCC | II | 105 | 15 |
| 5 | 65 | M | II | Right | 61 | 81 | 7x | - | 400 | 6 | BRC | - | 41 | 9 |
| 6 | 63 | F | IV | Left | 54 | 76 | 9p | 14 | 100 | 6 | ccRCC | I-II | 62 | 14 |
| 7 | 36 | M | II | Right | 38 | 84 | 4p | 11 | 20 | 5 | BRC | - | 80 | 6 |
| 8 | 36 | M | III | Right | 27 | 99 | 7a | 18 | 10 | 5 | BRC | - | 72 | 5 |
| 9 | 45 | M | III | Left | 41 | 96 | 9p | 14 | 300 | 3 | ccRCC | I | 53 | 4 |

Note: Pre-eGFR = Preoperative estimated glomerular filtration rate; ISUP = International Society of Urologic Pathology; Post-eGFR = Postoperative estimated glomerular filtration rate; ccRCC = clear cell renal cell carcinoma; PKD = polycystic kidney disease; BRC = benign renal cysts.
